# Spontaneous Iliopsoas Muscle Hemorrhage Secondary to Ibrutinib (Imbruvica; Pharmacyclics): Brief Report

**DOI:** 10.1177/2324709616648457

**Published:** 2016-05-09

**Authors:** Anna Sarcon, Gregory P. Botta, Nikunj Patel, Alan Saven

**Affiliations:** 1 Scripps Green Hospital, La Jolla, CA, USA

**Keywords:** hemorrhage, Imbruvica, iliopsoas muscle

## Abstract

Ibrutinib (Imbruvica; Pharmacyclics) is the first Food and Drug Administration–approved inhibitor of Burton’s tyrosine kinase (BTK). Attenuation of BTK signaling ultimately leads to inhibition of B-cell proliferation and apoptosis. After a series of clinical trials, the Food and Drug Administration approved ibrutinib in patients with relapsed chronic lymphocytic leukemia in 2014 and Waldenström’s macroglobulinemia in 2015. Those trials included rare grade 3+ hemorrhagic events associated with ibrutinib. Herein, we report a unique presentation of back pain due to iliopsoas muscle hemorrhage in a patient with Waldenström’s macroglobulinemia after initiation of ibrutinib.

## Introduction

Ibrutinib (Imbruvica; Pharmacyclics) is the first Food and Drug Administration–approved inhibitor of Burton’s tyrosine kinase (BTK). Attenuation of BTK signaling ultimately leads to inhibition of B-cell proliferation and apoptosis. After a series of clinical trials, the Food and Drug Administration approved ibrutinib in patients with relapsed chronic lymphocytic leukemia in 2014 and Waldenström’s macroglobulinemia in 2015.^1,2^ Those trials included rare grade 3+ hemorrhagic events associated with ibrutinib.

Herein, we report a unique presentation of back pain due to iliopsoas muscle hemorrhage in a patient with Waldenström’s macroglobulinemia after initiation of ibrutinib.

## Case Presentation

An 80-year-old male with history of demyelinating polyneuropathy, chronic hyponatremia, and Waldenström’s macroglobulinemia presented to the emergency room with 3-day history of swelling and pain in the right medial thigh region as well as lower back. He was initially diagnosed with Waldenström’s macroglobulinemia 8 years prior, after a bone marrow biopsy was performed in the setting of progressive anemia. Pathology revealed lymphoplasmacytic lymphoma comprising 30% of marrow cellularity, consistent with Waldenström’s macroglobulinemia. Initially, 4 doses of rituximab were prescribed and he received 1 cycle of bendamustine as well as 2 episodes of plasma exchange. Subsequently, he was initiated on 420 mg of ibrutinib 1 year prior to presentation and overall tolerated it well, only suffering a maculopapular rash of the extremities as well as mild lower extremity edema.

There was no clinical history of him consuming aspirin, nonsteroidal anti-inflammatory drugs, or any coagulation cascade inhibitors. There had been no trauma prior to presentation, and there was no history of spontaneous bleeding. On presentation to the emergency room, lower extremity ultrasound was performed, which was negative for deep vein thrombosis. Laboratory data revealed a hemoglobin of 12 g/dL (reference 13.5-17.0 g/dL), hematocrit of 36.4 (ref. 41-53), and platelet count of 159 k/mcl (ref. 150-450 k/mcl), which were all at baseline. Given the negative workup the patient was discharged home. He subsequently presented to the emergency room, 3 days later, with progressive severe right thigh and back pain. Laboratory data revealed hemoglobin of 9.1 g/dL, hematocrit of 25.8, platelets 171 k/mcl, prothrombin time of 10.3 seconds, and international normalized ratio of 1.0.

**Figure 1.**
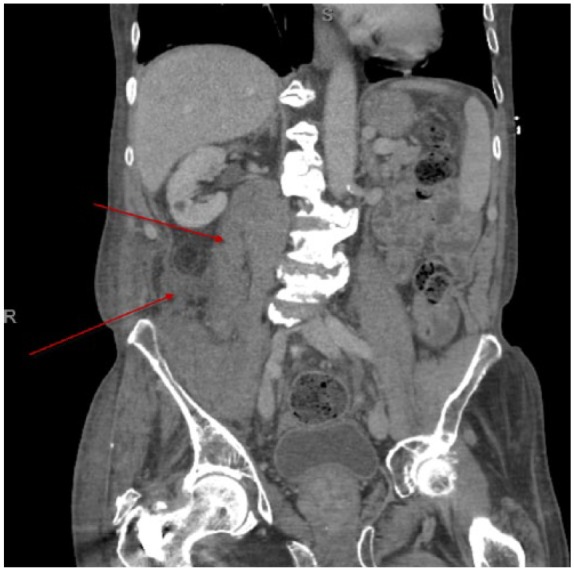
Top arrow, abnormal right iliopsoas–intramuscular hemorrhage; Lower arrow, hemorrhage in the retroperitoneum; contralateral, normal left iliopsoas.

Computed tomography of the abdomen and pelvis was obtained, which showed extensive intramuscular hemorrhage within the right iliopsoas musculature, extending from the level of the right renal pelvis into the right inguinal region, as well as a small amount of hemorrhage in the adjacent retroperitoneum (Figure 1). Computed tomography did not reveal any active bleeding however. The patient was admitted to the hospital for further care and stabilization. There was no evidence of femoral nerve compression and surgical intervention was not indicated. Furthermore, serial complete blood counts were obtained and his hemoglobin remained stable. Therefore, he was not transfused with any blood products. He was eventually discharged and ibrutinib was discontinued on diagnosis of iliopsoas hemorrhage. One-month follow-up showed stable hemoglobin, and the patient remains off ibrutinib due to ongoing concern about spontaneous hemorrhage.

## Discussion

We presented a case of retroperitoneal hemorrhage approximately 1 year after initiation of ibrutinib.

Presentation of retroperitoneal hemorrhage varies and can be rather nonspecific. Patients can have lower back, groin discomfort, and even hemodynamic instability. Hemorrhage in the iliopsoas muscle often leads to femoral neuropathy causing groin pain or leg weakness. The exact pathophysiology of spontaneous retroperitoneal bleeding is unclear. A retrospective study of 12 patients on anticoagulation with large rectus sheath hematoma revealed that 6 of these patients had a history of coughing fits.^3^ On the other hand, in patients with hemophilia, often a minor trauma can lead to spontaneous retroperitoneal bleeding.^4^ Without evidence of hemodynamic instability conservative therapy is recommended. This includes withdrawing the offending agent, correction of coagulopathy, and volume resuscitation.^5^

Common complications of ibrutinib have been reported in the literature, which include hemorrhage, infections, myelosuppression, renal toxicity, secondary primary malignancies, and embryo-fetal toxicity.6 Hemorrhage (including ecchymosis of any grade) has been observed in 63% of patients with chronic lymphocytic leukemia, of which 6% of cases included subdural hematoma, gastrointestinal bleeding, and hematuria.^1,2^ Due to complications associated with increased bleeding risk, withholding ibrutinib is recommended prior and after surgery by the manufacturer, Pharmacyclics.^6^

The initial RESONATE trial in 2014 found a significant improvement in progression-free survival when comparing ibrutinib to the anti-CD20 antibody ofatumumab at 9.4 months (end point not reached with ibrutinib vs 9.1 months with ofatumumab, respectively).^7^ Bleeding events (including petechiae and minor ecchymoses) were more common with ibrutinib compared with ofatumumab (44% vs 12%) while grade 3 or higher hemorrhage was reported in 2 patients taking ibrutinib (1%, one with a subdural hematoma) and 3 patients in the ofatumumab group (2%). The RESONATE-2 clinical trial found significant improvement in progression-free survival in the ibrutinib group versus standard of care (end point not reached with ibrutinib vs 18.9 months with chlorambucil). Specifically, 4 patients in the ibrutinib group had a grade 3 hemorrhage and one with grade 4 hemorrhage.^8^ Specifically, ibrutinib in Waldenström’s macroglobulinemia had a significant 100% overall response and 91.2% major response rate in MYD88^L265^PCXCR^WT^ patients.^9^ This study found grade 2 epistaxis-related bleeding in 2 patients (3% of study population). Furthermore, 2 other patients (3% of study population) had unspecified postprocedural bleeding that was attenuated with cessation of fish oil supplements.^9^

To our knowledge, no cases of retroperitoneal bleeding in patients with Waldenström’s macroglobulinemia treated with ibrutinib have been reported in the literature thus far. Clearly, risks and benefits of treatment initiation should be considered closely in patients.
